# Glucose lowering treatment in high fat diet‐induced cognitive impairment indicates that cognitive deficits may be glucose independent

**DOI:** 10.1002/alz70859_098593

**Published:** 2025-12-25

**Authors:** Sarah E Elzinga, Diana M Rigan, Crystal Pacut, Sam Teener, Ian Webber‐Davis, Andrew Carter, Haley McQuown, Mohamed Noureldein, Lillia Baird, Benjamin Murdock, Tahra Suhan, Bertram Pitt, Ahmed Abdel‐latif, Eva L Feldman

**Affiliations:** ^1^ Michigan State University, East Lansing, MI USA; ^2^ University of Michigan, Ann Arbor, MI USA

## Abstract

**Background:**

The rates of metabolic stress (obesity, prediabetes, metabolic syndrome, and diabetes) are on the rise. Metabolic stress (particularly in midlife) increases the risk of later life cognitive impairment, including dementias such as Alzheimer’s Disease and Alzheimer’s Disease Related Dementias (AD/ADRD). Targeting midlife metabolic stress is therefore a promising therapeutic approach to preventing later life AD/ADRD. Further, while sodium‐glucose cotransporters (SGLT) inhibitors can be used to lower glucose and reduce risk of cardiorenal outcomes in metabolic stress, evidence suggests that they may also have beneficial effects on the brain. However, their specific impact on the brain and on cognition, particularly when targeting both SGLT1 and 2, is not well known.

**Method:**

At 8wks of age, obesity, prediabetes, and hypertension were induced in male C57BL/6 mice by feeding high fat diet (HFD) and administering L‐Nitro_L‐arginine methylester (0.8 g/L) in drinking water. After 5 weeks on diet, control and HFD mice were assigned to either dual SGLT1 and 2 inhibitor treatment (Sotagliflozin (SOTA); 30 mg/kg) or vehicle given via daily oral gavage for 10wks. Prior to treatment and at terminal, mice were assessed for cognition, using puzzle box and/or Morris Water Maze. At terminal, metabolic testing (body weights, glucose tolerance, HbA1c) and inflammatory analyses (plasma cytokines) were also assessed.

**Result:**

SOTA treatment significantly decreased body weight and improved glucose tolerance in both HFD and control mice. At terminal, STG treated animals also had lower HbA1c levels, regardless of diet. Additionally, plasma CCL‐2 cytokine levels were elevated in HFD mice compared to controls and were significantly lowered with SOTA treatment. As expected, HFD worsened cognitive outcomes (prior to treatment). However, SOTA treatment improved executive function in controls while having no effect in HFD animals. Further, at terminal HFD animals had worse spatial learning and memory outcomes compared to controls, which were not altered with SOTA treatment.

**Conclusion:**

SOTA treatment improved metabolic outcomes regardless of diet. However, cognition was only improved in control animals, indicating that metabolic stress‐induced cognitive impairment may be glucose independent.

